# Editing *SOX* Genes by CRISPR-Cas: Current Insights and Future Perspectives

**DOI:** 10.3390/ijms222111321

**Published:** 2021-10-20

**Authors:** Ali Dehshahri, Alessio Biagioni, Hadi Bayat, E. Hui Clarissa Lee, Mohammad Hashemabadi, Hojjat Samareh Fekri, Ali Zarrabi, Reza Mohammadinejad, Alan Prem Kumar

**Affiliations:** 1 Center for Nanotechnology in Drug Delivery, Shiraz University of Medical Sciences, Shiraz 7146864685, Iran; dehshahria@sums.ac.ir; 2 Department of Experimental and Clinical Biomedical Sciences “Mario Serio”, University of Florence, 50134 Florence, Italy; alessio.biagioni@unifi.it; 3 Department of Tissue Engineering and Applied Cell Sciences, School of Advanced Technologies in Medicine, Shahid Beheshti University of Medical Sciences, Tehran 1985717443, Iran; h.bayat87@outlook.com; 4 Department of Molecular Genetics, Faculty of Biological Sciences, Tarbiat Modares University, Tehran 14115111, Iran; 5 Cancer Science Institute of Singapore, National University of Singapore, Singapore 637551, Singapore; e0032613@u.nus.edu; 6 Department of Pharmacology, Yong Loo Lin School of Medicine, National University of Singapore, Singapore 637551, Singapore; 7 Department of Biology, Faculty of Sciences, Shahid Bahonar University, Kerman 7616914111, Iran; mohammadhashemabadi@gmail.com; 8 Student Research Committee, Kerman University of Medical Sciences, Kerman 7619813159, Iran; hojjatfekri@gmail.com; 9 Pharmaceutics Research Center, Institute of Neuropharmacology, Kerman University of Medical Sciences, Kerman 7616911319, Iran; 10 Department of Biomedical Engineering, Faculty of Engineering and Natural Sciences, Istinye University, Sariyer, Istanbul 34396, Turkey; alizarrabi@gmail.com; 11 Neuroscience Research Center, Institute of Neuropharmacology, Kerman University of Medical Sciences, Kerman 7619813159, Iran; 12 NUS Centre for Cancer Research (N2CR), Yong Loo Lin School of Medicine, National University of Singapore, Singapore 637551, Singapore

**Keywords:** CRISPR, SOX transcription factors, gene editing, cancer, stem cells

## Abstract

Clustered Regularly Interspaced Short Palindromic Repeats (CRISPR) and its associated proteins (Cas) is an adaptive immune system in archaea and most bacteria. By repurposing these systems for use in eukaryote cells, a substantial revolution has arisen in the genome engineering field. In recent years, CRISPR-Cas technology was rapidly developed and different types of DNA or RNA sequence editors, gene activator or repressor, and epigenome modulators established. The versatility and feasibility of CRISPR-Cas technology has introduced this system as the most suitable tool for discovering and studying the mechanism of specific genes and also for generating appropriate cell and animal models. *SOX* genes play crucial roles in development processes and stemness. To elucidate the exact roles of SOX factors and their partners in tissue hemostasis and cell regeneration, generating appropriate in vitro and in vivo models is crucial. In line with these premises, CRISPR-Cas technology is a promising tool for studying different family members of SOX transcription factors. In this review, we aim to highlight the importance of CRISPR-Cas and summarize the applications of this novel, promising technology in studying and decoding the function of different members of the *SOX* gene family.

## 1. Introduction

### 1.1. CRISPR-Cas System

**Figure 1:**
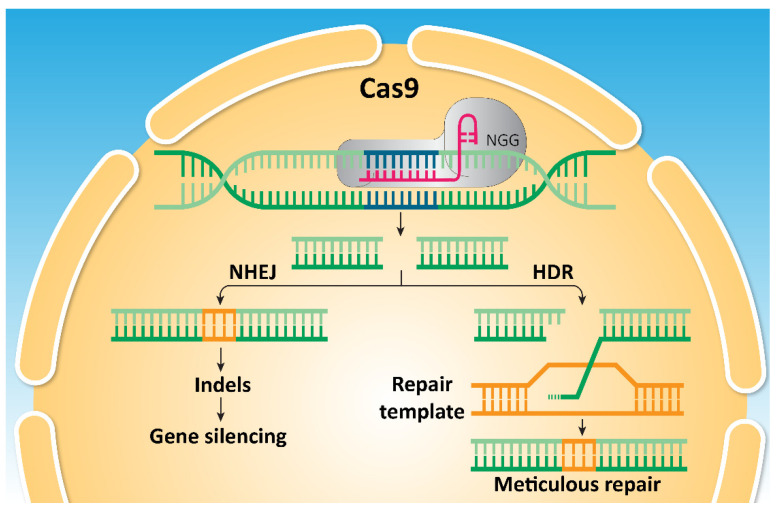
CRISPR-Cas9 mediated gene editing. A single Cas9 effector recognizes the target region and blunt-ended double-stranded breaks (DSBs) would be induced at target site through Cas9 endonuclease domains. The DSBs introduced by Cas9 endonuclease would promptly be repaired by the error-prone NHEJ pathway or by most specific HDR. The NHEJ pathway might result in random Indels and disrupt the sequence frame at the target site. Alternatively, when a repair template is supplied, the HDR pathway increases the accuracy and efficiency of the targeted gene editing.

The CRISPR-Cas is an adaptive immune system used by bacteria and archaea to protect themselves from foreign nucleic acids invasion, such as phages or plasmids [1,2,3]. In 2012, for the first time, the CRISPR-Cas system was demonstrated to be a promising genome editing tool to be used for selective gene manipulation in both in vitro and in vivo models (Figure 1) [4,5]. Compared to genome editing tools based on protein-DNA interaction such as meganucleases, ZFNs, and TALENs, the technology of CRISPR-Cas is more versatile and feasible because it relies on base pairing of nucleic acids; and the required guide RNA (gRNA), the complex of crRNA and tracrRNA could be easily engineered as a single transcript, avoiding the need for custom synthesis, purification, and validation of targeted DNA-binding proteins [5,6,7].

Native CRISPR-Cas systems are classified into two main classes and subcategorized into six types of Cas effector proteins. CRISPR-Cas9, classified into class-II and type II, is the most common system widely used in several CRISPR-based genome editing approaches [8]. Moreover, two other popular single effector Cas proteins in class-II are (i) Cas12a, which is subcategorized in type V and, unlike the natural Cas9, recognizes T-rich PAM, by a single gRNA [9,10] and (ii) Cas13, which is classified in type VI and is able to precisely target the RNA [11]. To date, several strategies are available to deliver the CRISPR-Cas machinery in vitro and in vivo.

The delivery systems are needed for biomedical applications of CRISPR-Cas [6,12]. Viral vectors, comprising adenoviruses, adeno-associated viruses (AAVs), and retroviruses, are very efficient delivery systems [12,13]. Moreover, viruses are extensively used for cancer therapy due to their ability to preferentially infect cancer cells in an active proliferative status, exploited in the commonly called oncolytic therapy [14]. Other less widely used methods are combining the exogenous form of the Cas9 with the selected gRNA, a formulation called ribonucleoproteins (RNPs), transferable into cells by lipid-mediated delivery [15], exosomes-derived vesicles [16], nano-formulations such as gold nanoparticles [17], copper sulfide nanoplatform [18], dendrimers [19], apoferritin [20], supramolecular polymers [21] or nanoclews [22]. The advantages of these nonviral delivery systems include low immunogenicity and expense, simple scalability, and safety [23,24,25,26,27,28,29,30,31,32,33]. All the above-described systems have expanded the possibilities to deeply study the molecular mechanisms and help decode unknown functions of several important cell proteins [34]. The *SOX* gene family are associated with several biological functions, from stemness to carcinogenesis, and by using CRISPR-Cas genome editing tools, the exact mechanism and function would be more reliably appreciated.

### 1.2. SOX Proteins

**Figure 2:**
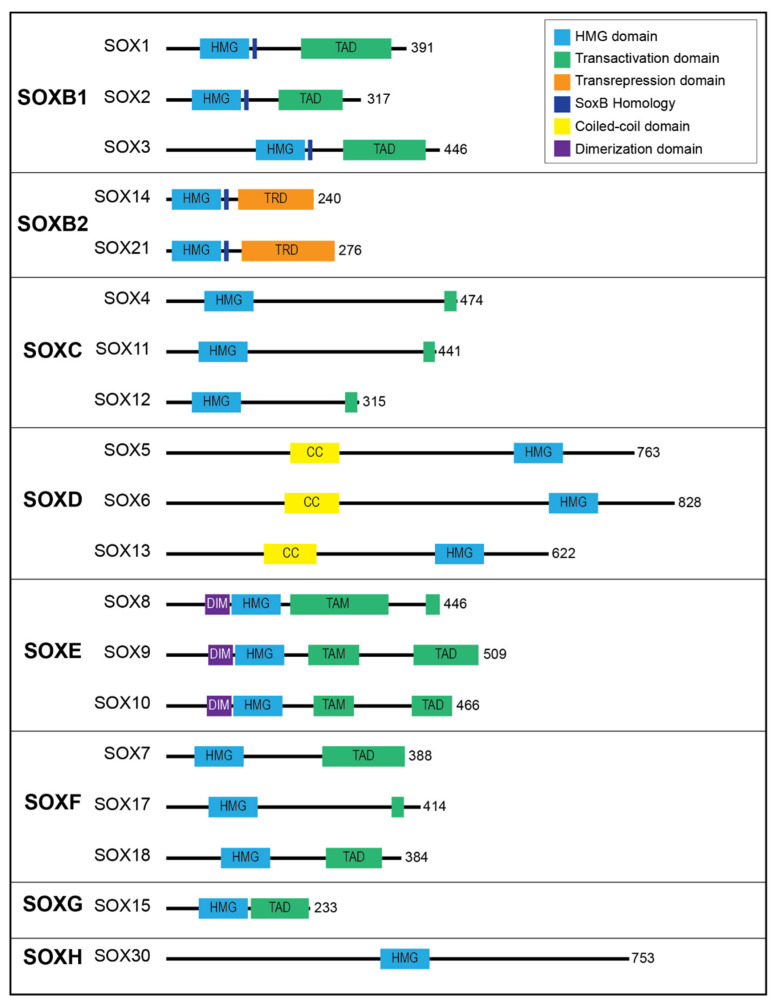
Subfamiles of SOX proteins and the functional domains. The major protein functional domains include the high-mobility group (HMG) domain, transactivation domain (TAD), coiled-coil (CC) domain, transrepression domain (TRD), dimerization (DIM) domain, and SoxB homology domain. Reprinted by permission from Frontiers, Frontiers in Physiology [42], Copyright 2020.

The SOX (SRY homology box) proteins affect stem cell function and fate by regulating the expression of genes involved in self-renewal and multipotency [35,36,37]. These proteins are overexpressed in many different tumors [38]. SOX proteins are able to bind to the DNA sequence motif ATTGTT, trigger conformational changes, and bend DNA specifically [39]. These proteins as ‘pioneer’ factors recruiting non-pioneer transcription factor (TFs) drive cell fate conversions [40]. This protein family includes 20 members, which mainly share a conserved DNA-binding element HMG domain, a transcriptional master regulator of virility [41]. SOX proteins as lineage-associated TFs are classified into different categories (from SOXA to H) according to homology within the HMG domain (Figure 2) [42].

Post-translation modifications of SOX proteins including phosphorylation, methylation, ubiquitylation, acetylation, and SUMOylation have been reported and represent an annotated function. Therefore, targeting the enzymes that catalyze these modifications may affect therapeutic strategies for human diseases [43]. Some published review articles have discussed the diverse functions of SOX proteins across cancer, stem cells, and development [35,44,45,46]. For example, SOX8 was a master regulator for sense organ cell reprogramming [47] and SOX15 determined as a oocyte-enriched reprogramming factor [48].

**Figure 3:**
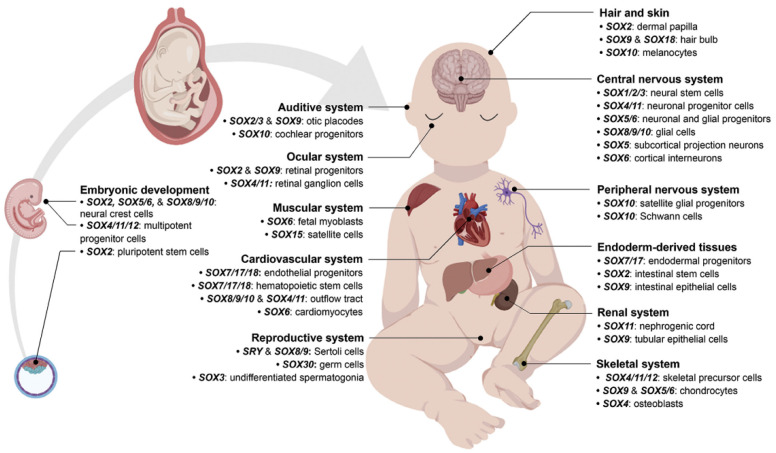
Developmental aspects of SOX protein subfamilies. *SOX* genes play crucial functions in hair, skin, eye, auditive system, musculoskeletal system, cardiovascular system, nervous system, gastrointestinal system, reproductive system, and embryonic development. Reprinted by permission from Elsevier, Trends in Genetics [49], Copyright 2019.

**Figure 4:**
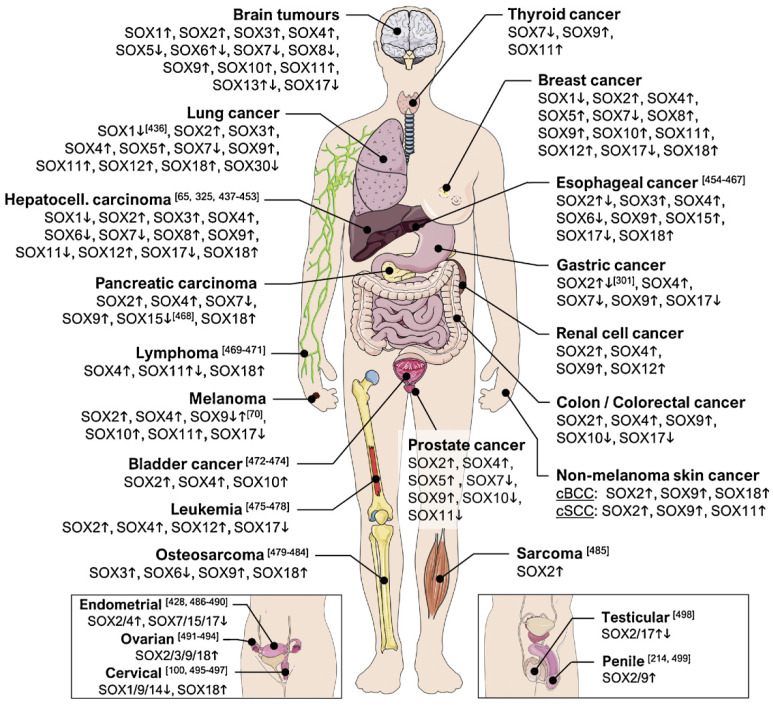
The involvement of SOX protein subfamiles in human cancers. *SOX* genes can be upregulated or downregulated in tumor cells including skin cancer, bone cancer, brain cancers, respiratory cancers, gastrointestinal cancers, leukemia, urological cancers, gynecologic cancers, and breast cancer. Reprinted by permission from Elsevier, Seminars in Cancer Biology [46], Copyright 2020.

In this review, we aim to summarize recent advancements in studying different members of the *SOX* gene family by using CRISPR-Cas genome editing tools. The versatility and feasibility of CRISPR technology introduced this system as a promising tool for uncovering unknown mechanisms and drawing reliable signaling pathways. *SOX* genes are extremely important factors, especially during development (Figure 3) [49] and cancer initiation, progression, invasiveness, and metastasis (Figure 4) [46,50]. Compared to other genome editing tools, CRISPR-Cas technology is easily programmable, cost-effective, and could be efficiently applied to study different *SOX* genes and treat SOXopathies [49].

## 2. SOX2 Involvement in Cancer and Stem Cell Fates

SOX2 is currently the most studied member of the large SOX family, often interacting with a series of co-factors such as OCT3/4 and PAX6 [51,52] through its C-terminus domain, while its N-terminus, having the HMG-domain, the nuclear localization sequence and the nuclear export sequence, plays a fundamental role in the subcellular distribution [53,54]. The HMG domain in particular has been characterized in deep and a DNA recognition consensus has been defined for SOX2 (i.e., CCCATTGTTC in man and CTTTGTC in mouse) [40,55]. Although the TTGT element is the preferred recognition motif for all SOX proteins, SOX2 is able to keep in contact with several transcription factors in an unspecific and promiscuous manner [56]. It is interesting to note that SOX2 is able to up-regulate itself and exploit an autoregulatory feed-forward mechanism [57]. Interestingly, the entire *SOX2* gene further falls into the intron of a much greater product, which is called SOX2OT (SOX2 overlapping transcript) [58]. Indeed, when planning a *SOX2* knock-out (KO) experiment, it should be taken under consideration that every *SOX2* manipulation obviously involves potential side-effects on SOX2OT [59]. SOX2OT plays an important role in carcinogenesis by promoting tumor cell proliferation, invasion, migration, and growth and suppressing apoptosis mainly through the regulation of some cancer stem cell (CSC) factors such as OCT4, NANOG, ALDH1, CD44, and CD133 [60,61]. Moreover, it was established that SOX2OT is also capable to bind miR-200 family members to regulate *SOX2* expression. SOX2 is not the only member of the SOX family regulated by SOX2OT as it was reported that it is able to modulate the mRNA and protein expression of *SOX3* as well [62]. SOX2OT was demonstrated to promote epithelial-mesenchymal transition and stemness in several kinds of cancer cells [63,64,65]. SOX2OT is also involved in the inhibition of the JAK/STAT signaling pathway [62]. While two gene-proximal enhancers, SOX2 regulatory region 1 (SRR1) and SRR2 were previously described to act as cis-regulator for *SOX2* expression [66], Zhou et al. identified three novel enhancers, i.e., SRR18, SRR107, and SRR111, which form a chromatin complex with the *SOX2* promoter in embryonic stem cells (ESCs) [67]. Moreover, a 13 kb-long super-enhancer was described to be located 100 kb downstream of SOX2 in mouse ESCs, which may interact with OCT4, SOX2, and Nanog as trans-acting factors to enhance SOX2 expression via DNA looping. Li et al. used a double-CRISPR genome editing approach to delete the entire super-enhancer sequence and demonstrate that it is responsible for over 90% of *SOX2* expression [68]. Deleting the core of the CCCTC-binding factor (CTCF) binding site by CRISPR-Cas9 editing tool in the SOX2 super-enhancer resulted in cohesin recruitment loss and disrupting the formation of chromatin loops, which in turn reduced SOX2 expression [69].

**Figure 5:**
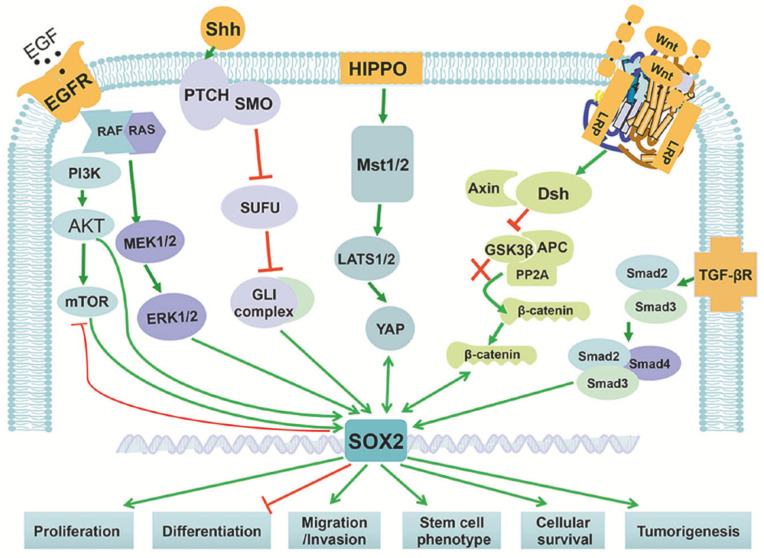
SOX2 cross-talks with various cellular pathways. SOX2 cross-talks with some signaling pathways including TGF-β, WNT, HIPPO, Hedgehog, and EGFR regulates cell proliferation, survival, migration, and differentiation. Reprinted by permission from Springer Nature, Signal Transduction and Targeted Therapy [76], Copyright 2020.

SOX2 is widely known as a master orchestrator in all reprogramming applications. Moreover, SOX2 collaborates with several co-factors such as OCT4, KLF4, and cMYC [70] and enables the derivation of human or murine-induced pluripotent stem cells (iPSCs) from terminally differentiated somatic cells. Such phenomenon was partially observed in vivo, where SOX2 plays key roles in the stem potential of the inner cell mass of the blastocyst [71] and neural cell lineages formation [72]. Moreover, SOX2 is orchestrating the development of the gastrointestinal tract, where its expression is associated with the engulfing foregut and derived endodermal structures from which the esophagus and anterior stomach evolve [73]. SOX2 balancing is important for tissue homeostasis and therefore its aberrant expression is often associated with various forms of cancers [74]. In fact, SOX2 induces the acquisition of stem-like features and in some cases, generates CSCs (Figure 5) [75].

In particular, SOX2 induction has been reported at early stages in breast or ovarian cancers and is associated with disease progression, metastasis, and relapse [77,78]. A high expression level of *SOX2* is associated with increased cell motility and metastasis in glioblastoma [79]. Whereas in gastric cancer, *Helicobacter pylori* infections have influenced *SOX2* expression, activated PTEN, and consequently inhibited PI3K/AKT-driven cell cycle progression and apoptosis [80]. Moreover, it is elucidated that microenvironmental factors might play an important role in *SOX2* regulation in cancer [81]. Indeed, extracellular acidosis was demonstrated to increase *SOX2* expression in melanoma, confirming that SOX2 is also able to influence cancer cell metabolism profile to a more oxidative phenotype through the hypoxia-inducible factor 1-α (HIF1α) pathway [74]. SOX2, which is induced by an acidic microenvironment, was demonstrated to enhance several OxPhos-related genes and thus, its depletion led to a more glycolytic profile, negatively regulating PGC1α and inducing the switch of MCT genes from type 1 to type 4. Indeed, chemoresistant cancer cells, often characterized by high levels of SOX2, are commonly more prone to exploit the oxidative metabolism [82]. In normoxia, HIF1α and SOX2 are inversely correlated, reprogramming cancer cells towards an OxPhos profile, while under hypoxic conditions, as well as in acidosis-exposed cells, the increased lactate production may promote HIF1α stabilization reducing PGC1α towards a glycolytic re-conversion. Indeed, such a metabolic switch is often associated with enhanced drug resistance and metastatic ability [83,84]. By emerging CRISPR-Cas9 genome editing technology, several research lines have been established to better understand the importance of SOX2 in cell differentiation, the acquisition of stem characteristics, and tumor progression (Table 1) [85,86,87].

### SOX2 Gene Editing Mediated by CRISPR

**Figure 6:**
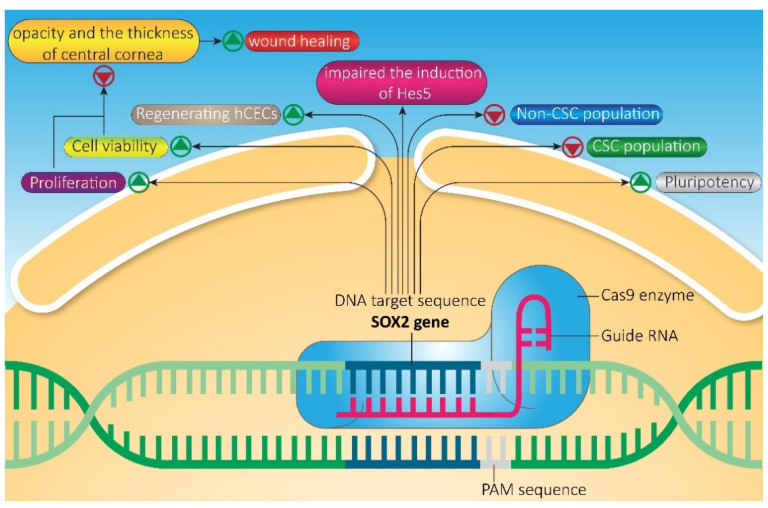
Applications of the CRISPR system to edit *SOX2*. Editing *SOX2* by CRISPR-Cas affects cell viability, proliferation, pluripotency, and tissue regeneration.

SOX2 plays a major role in both tumorigenesis and embryogenesis, especially during the development and differentiation of the neuroectodermal layer. Yang et al. demonstrated that CRISPR-Cas9-mediated KO of ATF1 significantly up-regulates neuroectoderm genes, *SOX2* and *PAX6*, in human embryonic stem cells (hESCs). However, the overexpression of ATF1 suppressed neuroectodermal differentiation. In line with these premises, they indicated that SOX2 induction is pivotal for the up-regulation of PAX6 and SOX1, and introduced ATF1 as a negative regulator for *SOX2* expression [88]. Cheng et al. exploited the PC transposon system to KO SOX2 in neural progenitor cells by in utero electroporation (IUE) with promising results. Indeed, SOX2 depleted as early as three days post-IUE, whilst expressions of SOX1 and PAX6 remained intact, demonstrating no off-target effects [89]. Moreover, they also proved that both the wild-type Cas9 and the Cas9n exert the gene-editing with comparable KO efficiency. It is also elucidated that knocking out SOX2 impaired the induction of the neural progenitor gene, *Hes5*, in mouse and chick embryos and the subsequent commitment to the neuronal lineage. In fact, SOX2 promotes the neurogenic domain formation in the nasal epithelium, establishes, maintains, and expands the neuronal progenitor pool by decreasing Bmp4 and up-regulating Hes5 expression. Therefore, SOX2 acts as a negative regulator for Bmp4 expression [90]. As both the neural crest and derma originated from the ectodermal layer, in some cases, brain tumors and melanoma share several features. However, melanoma cells mainly have a high expression level of SOX2 while it is not observed in neural crest stem cells [91]. To date, the role of SOX2 has been controversial in melanoma. Although it is reported that SOX2 may start the tumor initiation process via CDK1 in melanoma [92], knocking-out of *SOX2* by CRISPR-Cas9 does not affect melanoma progression and metastasis [91]. Maurizi et al. demonstrated that SOX2 is required for osteosarcoma initiation and development in a mouse tumor model and is essential for survival and proliferation. They indicated that *SOX2* inhibition by CRISPR-Cas9 in osteosarcoma cells decreases viability and proliferation of both CSC and non-CSC populations. Furthermore, it is indicated that the overexpression of YAP rescues cells from the lethality caused by SOX2 inactivation [93]. The loss of SOX2 is sufficient to maintain a seminoma-cell fate of seminomatous TCam-2 cells and after in vivo injection for about six weeks, these cells have been reprogrammed to an embryonal-like status. Moreover, knocking-out of FOXA2 strengthened such effect up to 12 weeks [94]. CRISPR/Cas9 was also exploited to generate a luciferase knock-in (KI) system under the control of the *SOX2* promoter in HEK293T cells, demonstrating to be a novel and useful tool to study the transcriptional regulation of *SOX2* [89]. Similarly, Balboa et al. provided a fluorescent marker of SOX2 endogenous expression through the knocking-in of a T2A fused nuclear tdTomato reporter before the stop codon of the *SOX2* gene coding sequence by CRISPR-SaCas9 [95]. To date, many fluorescent systems are available to evaluate SOX2 endogenous expression rapidly and in a real-time manner in several cell lines to better understand how it is spatially and temporally regulated during embryogenesis and neural differentiation [96]. Yang et al. generated mice with a tag or a fluorescent reporter construct in *Nanog*, *SOX2*, and *OCT4* genes through a one-step procedure by co-injection of gRNAs and Cas9 mRNA directly into zygotes, demonstrating that with such methodology the risk of off-target mutation is significantly reduced [97]. Mei et al. exploited CRISPR-dead Cas9 (dCas9) system to induce Yamanaka’s factors (OCT4, SOX2, KLF4, and MYC) in a luminal breast cancer cell line with an innovative multiplexing system. Indeed, they designed particular tRNA-gRNA architecture in order to allow the endogenous cell tRNA-processing system to precisely cleave both ends of the tRNA precursor and thus release the gRNAs. Such an approach led them to gain a stable cell strain characterized by increased invasion, proliferation and stemness features, with a similar drug response pattern of HER2 positive cells [98]. Moreover, Chang et al. used CRISPR-dCas9 to induce *SOX2* expression in the rat cornea. They reported that the activation of *SOX2* reduces the opacity and the thickness of the central cornea by increasing cell viability and proliferation of corneal endothelial cells, which normally are not able to regenerate after a wound or a disease (Figure 6) [99].

## 3. SOX2/3 Contribution in Regenerative Medicine

Of particular interest is the CRISPR application on the little salamander, called Axolotl (*Ambystoma mexicanum*), which is to date the only tetrapod that functionally regenerates all cell types of the limb and spinal cord. For this reason, it represents an important animal model for regenerative medicine studies [103]. Fei et al. exploited both TALEN and CRISPR technologies to KO *SOX2* and demonstrated that its expression is the key for spinal cord regeneration while it did not affect larval viability and development. On the other hand, they also indicated that *SOX3* expression is fundamental during development, but not for regeneration after tail amputation [100]. By using the HDR repair pathway via CRISPR-Cas9, they also successfully inserted a fluorescent reporter gene and a larger membrane-tagged Cherry-ERT2-Cre-ERT2 cassette into SOX2 and Pax7 genomic loci in the axolotl animal model. In these regards, it was elucidated that PAX7-positive satellite cells are the major contributing source in the myogenesis process during axolotl limb regeneration, while SOX2-positive cells are mainly located in the central nervous system, the lens, the head/tail lateral line neuromasts, and the spinal cord [104]. Knocking-out of *SOX2* has also been performed by in vivo injection of Cas9 protein–gRNA complexes into the spinal cord lumen of the axolotl, with subsequent electroporation [105]. Such an approach was incredibly efficient through protecting Cas9 from typical RNase activity in the cerebral spinal fluid, which normally prevents the electroporation of unprotected mRNAs [106]. The major challenge using human pluripotent stem cells in regenerative therapy is the risk of teratoma formation due to contamination of undifferentiated stem cells. To overcome this limitation, using a suicide gene for killing undifferentiated stem cells seems a promising strategy to provide a safety control before transplantation of stem cell-derived products. Hence, Wu et al. used the CRISPR-Cas9 editing tool to KI the iC9 suicide gene into the endogenous *SOX2* locus in the hESC-H1. With this strategy, they demonstrated that undifferentiated H1-iC9 cells were committed to apoptosis by iC9 inducer AP1903, whilst differentiated cell lineages including hematopoietic cells, neurons, and islet beta-like cells were not affected [101].

## 4. *SOX9* Gene Editing Mediated by CRISPR

SOX9 has shown a great impact on the specification, differentiation, and maintenance of various cell types during cell development [107,108]. Various developmental processes such as sex determination, pancreas development, and chondrogenesis are associated with the expression of *SOX9*. Several studies reported the pathological consequences of *SOX9* mutations. For example, it has been shown that the *SOX9* mutation leads to campomelic dysplasia. This disorder is characterized by bowed and shortened long bones, the bell-shaped thorax, and respiratory distress. Abnormal SOX9 is often accompanied by sexual disorders [109,110,111]. In most mammals, SRY, a key factor for sex determination, up-regulates *SOX9*, which is important for testis formation [112,113]. It has been shown that testis-specific enhancer of SOX9 core (TESCO) acts as SOX9 enhancer and provides the binding site for SRY. In addition to TESCO, several other enhancers are associated with sex determination in mammals [114,115]. Totally, transcriptional activator SOX9 has been studied in several investigations and its importance has been demonstrated by different approaches such as CRISPR-Cas9 tools. It is revealed that far upstream of *SOX9*, there is a specific sequence, which is named the XY sex reversal region (XYSR). In order to identify a responsible sequence in XYSR, Ogawa et al. employed the CRISPR-Cas9 system to generate mutant mice with different deletions in XYSR. When the whole or partial sequence of XYSR was deleted, male to female sex reversal occurred. Interestingly, this sequence includes a gonad enhancer for *SOX9*. This study showed the application of CRISPR-Cas9 system for identification of critical sequences in sex determination via SOX9 [116]. In addition to TESCO, the role of a 3.2 kb testis specific enhancer of SOX9 (TES) in sex determination has been shown in several studies. Using CRISPR-Cas9 gene editing system for deleting TES or TESCO in XY fetal gonads, a reduction in *SOX9* expression level was observed. The results of this study indicated that TES and TESCO are substantial elements for regulation of *SOX9* transcription levels. However, the results showed that these elements are not the sole factors involved in sex determination via SOX9 [115].

The application of the CRISPR-Cas9 gene-editing platform is not limited to understanding the mechanism of sex determination through SOX9. There are several reports indicating that SOX9 is a gastrointestinal stem cell marker with oncogenic properties in tumor development. The up-regulation of SOX9 has been reported in various premalignant tumors [117,118]. On the other hand, the Hippo signaling pathway and YAP1, as its co-activator, were reported to play a significant role in the development of gastric cancer. It has been demonstrated that the transcription of SOX9 is regulated by the interaction between YAP1 and TEAD proteins at the *SOX9* promoter, which leads to the induction of CSC properties [119]. Also, there are several reports on the role of peroxisome proliferator-activated receptors (PPARs) in gastric cancer progression [120]. Knocking down of PPARδ in cell line models decreased the formation of tumorspheres as well as invasion via the reduction of *SOX9* expression. Disrupting YAP1 or *SOX9* by CRISPR-Cas9 gene-editing platform reduced PPARδ-mediated oncogenic functions. In other words, the poor clinical outcome of gastric cancer patients is correlated with high levels of YAP1 or SOX9. Furthermore, the transcription of *SOX9* is promoted by the formation of the PPARδ/YAP1 complex, which was shown by the CRISPR-Cas9 gene-editing system. [121].

SOX2-dependent activation of Wnt signaling in tamoxifen-resistant breast cancer cells leads to the increase of CSC content. Moreover, it has been shown that high levels of SOX9 are associated with shorter survival and poor clinical outcome in breast cancer patients [122,123]. In a study conducted by Domenici et al., the growth of tamoxifen-resistant breast tumors in an in vivo model reduced after knocking out of *SOX9* by using CRISPR-Cas technology. According to the results of this study, SOX2–SOX9 signaling axis can act as a key factor in controlling the luminal progenitor cell content and is essential for the activity of Wnt signaling pathway. Knocking out of *SOX9* by the CRISPR-Cas system has represented it as a potential therapeutic target in breast cancer [124].

Another application of CRISPR-Cas systems is the generation of various differentiated cells from human pluripotent stem cells. The differentiated cells could be generated from various cells, including ESCs and iPSCs [125]. It is indicated that astrocytes can rapidly (in 4 to 7 weeks, while conventional methods take 3 to 6 months) be generated from hESCs when the expression level of transcription factors including NFIA or NFIA plus SOX9 had been elevated by using CRISPR-Cas9 technology. This simple and fast method has provided the great opportunity to investigate the biological properties of astrocytes as well as their role in several diseases processes [125,126].

## 5. CRISPR-Cas Editing of Other *SOX* Genes

It has been shown that the pluripotency and differentiation of ESCs are highly dependent on RYBP (Ring1 and YY1 Binding Protein). Differentiation of ESCs to myocardial and neural cells could be disrupted by depletion of RYBP. To investigate the role of RYBP in neural differentiation, CRISPR-Cas9 genome editing technology was employed to generate an RYBP homozygous KO murine ESCs containing SOX1-GFP reporter. The generated cell line could be used for the investigation of the ESCs differentiation into neurons as well as the study of molecular mechanisms of neurogenesis and drug screening [127].

The role of *SOX2* and *SOX3* genes in the development of mouse testes and brain has been investigated to show their functional equivalency [128]. By using the CRISPR-Cas9 system, these two genes were mutated to demonstrate their functions are identical or different. The replacement of *SOX3* with *SOX2* revealed that the increased expression of *SOX2* functionally rescues the defects related to the depletion of SOX3 in the development of pituitary and testes and restores phenotypes associated with SOX3-null mice. These results demonstrated the equivalent functions of SOX2 and SOX3 for brain and testis development [128]. Likewise, for studying the role of SOX2 and SOX3 in otic/epibranchial placode induction, Gou et al. used CRISPR-Cas9 technology to generate mutant alleles of *SOX2* and *SOX3*. Their results demonstrated redundant functions of these genes in the production of otic and epibranchial tissue [129]. Moreover, this investigation also elucidated that the loss of SOX3 could be replaced by *SOX2* expression to rescue placodal deficiencies. The use of the CRISPR-Cas9 system in a zebrafish model revealed the cooperation of SOX2 and SOX3 in the regulation of otic/epibranchial placode induction [129].

However, the role of SOX3 is not limited to the brain, testis, or otic/epibranchial placode induction. Hong et al. investigated the molecular mechanism of folliculogenesis for the generation of female gametes. They generated *SOX3* KO zebrafish lines by using the CRISPR-Cas9 system to investigate the pathways involved in ovarian steroidogenesis and apoptosis. The involvement of SOX3 in these cellular processes was shown in SOX^−/−^ ovaries by up-regulation of apoptotic pathways in such cells while ovarian steroidogenesis was down-regulated [130]. *SOX3* KO also resulted in the retardation of follicle development. Moreover, it is indicated that SOX3 could bind to the promoter of cyp19a1a and enhance 17β-estradiol synthesis, which in turn prohibits apoptosis in follicle development [130].

Another interesting member of the SOX family is SOX4, which has shown a great impact on cellular development and differentiation. SOX4 has demonstrated significant transcriptional activation roles as well as suppression functions alone or together with other transcription factors. Several reports indicated the importance of *SOX4* expression in bladder cancer development. However, the exact role of SOX4 in bladder cancer tumorigenesis had not been elucidated. To find SOX4-regulated genes in the progression of bladder cancer, the CRISPR interference (CRISPRi) method was employed to suppress the expression of *SOX4* in specific bladder cancer cell lines. When the expression of *SOX4* was restored by using lentiviral vectors, the targeted cells were rescued, and this experiment showed the pivotal role of SOX4 in tumorigenesis. On one hand, restoration of *SOX4* expression increased invasiveness of cancer cells whereas this characteristic was decreased in *SOX4* KO cells. On the other hand, proliferation and migration properties did not change significantly in these cells, showing that SOX4 has no notable impact on these processes. Furthermore, gene expression profiling elucidated that there is a negative correlation between SOX4 and WNT5a expression levels, suggesting that low expression levels of *SOX4* result in higher levels of WNT5a, which is associated with decreased invasion phenotype. Therefore, it could be concluded that the invasion of bladder cancer cells is regulated by SOX4 through the repression of WNT5a [131].

SOX6 is another member of the SOX family, which has shown a substantial role in β-thalassemia. Red blood cell destruction could be decreased by the reactivation of fetal γ-globin. SOX6, as a negative regulator, binds to the γ-globin promoter, and silences the expression of fetal hemoglobin. Therefore, reactivation of γ-globin could be considered as a therapeutic approach to ameliorate the symptoms of β-thalassemia [132,133,134]. Silencing *SOX6* expression by using CRISPR-Cas9 technology revealed that γ-globin mRNA level increased. This finding indicated that inhibiting *SOX6* expression by using CRISPR-Cas9 technology could be considered as a therapeutic strategy for the treatment of β-thalassemia patients [135].

Great attention has been directed to SOX10 due to its important roles in the inner ear and embryonic development, particularly in neural crest cells and neural crest derivatives such as melanocytes [136]. Several reports indicated that *SOX10* mutations have various characteristic phenotypes, including severe hearing loss and pigmentary disturbance [137,138]. In literature, it is revealed that mutation in *SOX10* orthologous has an association with Waardenburg diseases. CRISPR-Cas technologies have shown promising improvements in generating animal models, which are extremely valuable in studying human unknown mutations. It is indicated that CRISPR-Cas9 technology led to successful results to generate gene-modified pigs harboring precise genetic mutation [139]. The chick embryo provides a great opportunity for such experiments due to the low cost and ease of manipulation. In this line, to KO *SOX10* as a key transcription factor in neural crest development, CRISPR-Cas9 technology was employed to silence this gene in the early chick embryo. The successful loss-of-function in chick embryos could be used in several developmental processes, including dissection of gene regulatory interactions [140]. Neural crest stem cells have a great potential for differentiation into various cell types. Since SOX10 is produced in early neural crest progenitors, the CRISPR-Cas9 genome editing tool was used to generate SOX10-Nano-lantern (NL) reporter hiPSCs. In this investigation, neural crest cells were purified from hips with an NL KI reporter. Unlike conventional SOX10-reporter lines, these cells achieved bicistronic expression of NL and *SOX10* gene. The *SOX10*-expressing cells showed self-renewal properties as well as great potential for differentiation into neural crest derivatives [141]. In another study, a non-disruptive *SOX10* KI reporter was generated using the CRISPR-Cas9 genome editing tool in rat ESCs to generate both in vitro and in vivo reporter models. Recently, it was indicated that SOX10 has the potential for visualization and isolation of precursor and mature oligodendrocytes from postnatal animals. On the other hand, rats have shown several advantages rather than transgenic mouse lines due to the relative ease of surgical procedures and superiority of demyelinating lesions in rat models [142,143]. In addition, rat models are more suitable for cognition assays. The successful germline transmission provided a platform to generate animal models [144].

SOX10 has been considered as an oligodendrocyte lineage master regulator gene that could be used for reprogramming fibroblast cells to oligodendrocyte progenitor-like cells. In an investigation conducted by Matjusaitis et al., SOX10 along with Olig2, and Nkx6-2 were delivered to the target cells to enhance the differentiation of neural stem cells. Delivery of these three key oligodendrocyte lineage master regulatory genes resulted in reprogramming mouse embryonic fibroblasts to oligodendrocyte progenitor-like cells [145].

The association of the SOX family with the ability of the peripheral nervous system (PNS) for regeneration following injury has been shown via activation of the transcription factor SOX11 [146]. Perry et al. also confirmed that a regulatory network orchestrates the regeneration program in PNS following injury [147]. The regeneration-associated genes (RAGs) are members of the transcriptional response to injury and result in the synthesis of adhesion molecules and neuropeptides as well as cytoskeletal elements and cytokines [148]. The most important RAGs include transcription factors such as Jun, Atf3, and SOX11. Moreover, it is found that long noncoding RNAs (lncRNAs) play a key role in the regeneration of neurons. It is demonstrated that one of the lncRNAs, Silc1, led to neuroregeneration via the activation of *SOX11* [147].

The other member of the SOX family is SOX17, which has shown a great impact on endoderm development. In developmental biology studies, mouse models are commonly used to study the function and molecular mechanism of specific genes in developmental processes. In a study carried out by Suzuki et al., CRISPR-Cas9 technology was used to generate SOX17-2AEGFP (endoderm marker), Otx2-2A-tdTomato (ectoderm marker), and T-2A-TagBFP (mesoderm marker) bicistronic reporter KI mouse models. These mouse models enable researchers to visualize the endodermal, ectodermal, and mesodermal tissues during gastrulation [149].

Two genome-wide association studies and a meta-analysis analysis were carried out to identify the genetic determinants of risk in pulmonary arterial hypertension (PAH). The results revealed that the risk variants near SOX17 change gene regulation through a lineage-specific enhancer, which is active in endothelial cells. When this enhancer was inactivated by using CRISPR-Cas9 technology, a significant reduction in *SOX17* expression was observed. These results confirmed the association of genetic variation in the enhancer near *SOX17* with PAH. In this line, more attention is needed to find the impairment of SOX17 function that results from the genetic variation at loci the enhancer near *SOX17*.

Reduced fertility on female mice has been reported following the haploinsufficiency of SOX17. Also, infertility has been observed in mice with ablation of SOX17 in progesterone receptor promoter (Pgr)-positive cells due to the lack of uterine glandular structures [150]. Based on several investigations, SOX17 acts as a downstream target of the Pgr-Gata2-dependent transcription network. Wang et al. reported that ablation of SOX17 could impair leukemia inhibitory factor and Indian hedgehog homolog (IHH) signaling pathway and result in embryo implantation failure. Deleting SOX17-binding region, 19 kb upstream of the Ihh locus, by using CRISPR-Cas technology reduced Ihh expression which in turn resulted in the pregnancy impairment. These results showed that SOX17 regulates endometrial epithelial-stromal interactions and acts as a key regulatory element necessary for endometrial epithelial gene expression (Table 2) [151].

## 6. Conclusions

Genome editing tools during recent years have had a great impact on scientific research and therapeutic approaches. The CRISPR-Cas systems, winner of Nobel Prize in 2020, are the most groundbreaking technology in the field of genome editing and have revolutionized the insights towards establishing novel therapies for improving human health. By utilizing CRISPR-Cas systems, we can easily introduce various kinds of modifications such as targeted editing of DNA or RNA sequence, up-regulating or down-regulating specific genes, and even reprogramming epigenetic status in target cells. CRISPR-Cas-based tools have emerged as a powerful gene modulator mostly in in vitro studies. In this line, it would be extremely crucial to evaluate the efficiency of CRISPR-based tools in different in vivo applications. Currently, there are several crucial challenges, including off-target effects and promising methods for delivering the CRISPR-Cas system into target cells that need to be addressed precisely. Delivering the RNP form of the CRISPR-Cas system has shown promising results rather than plasmid DNA or mRNA. Recently, synthetic nanoparticles have been used broadly for delivering CRISPR-Cas systems in both in vitro and in vivo studies. However, it seems urgent that long-term studies should be performed to validate the safety of the components which are utilized in these cases. Recently, it was reported that the CRISPR-Cas clinical trial was successful for sickle cell disease and β-thalassemia. This finding could bring promising hope for ex vivo gene editing strategies and their use in clinical trial approaches [156]. CRISPR-Cas systems are powerful tools for discovering and studying genes such as the SOX family of transcription factors. SOX factors play important roles in development and stem cell biology. Generating appropriate cell and animal models gives the opportunity to address fundamental questions about the roles of SOX factors in the development process compared with their impact on tissue homeostasis and regeneration. The versatility and feasibility of CRISPR-Cas systems help the researchers to use these tools in studying the mechanisms of SOX factors in biological processes. The combination of CRISPR-based screening systems with available expression and ChIP-seq data could discover unknown partners for SOX factors that would help to generate more appropriate models and cell lineages from cultured pluripotent or differentiated cells. SOX factors such as SOX17 and SOX9 seem also play supporting roles in initiating human cancers by providing primitive stem-cell-like states. CRISPR-Cas systems could be applied for decoding the real function of SOX factors in human cancers as well.

In summary, CRISPR-Cas tools have revolutionized research and therapeutic approaches in the field of genome engineering. During recent years, CRISPR-based tools have shown promising potential for use in discovering and studying the mechanisms of human genes. *SOX* genes are pivotal factors during development processes in stem cell biology. By using CRISPR-Cas technologies, the exact function of SOX factors in these processes could be elucidated and might lead to establish novel therapeutic strategies for human diseases and cancers.

## Figures and Tables

**Table 1:** CRISPR targeting *SOX2*.

Cell Line/Animal Name	KO/KI	Outcome	Refs
TCam-2 Cell line	KO	Maintaining a seminoma-cell fate in vivo for about six weeks	[94]
Human corneal endothelial cells (hCECs)	Activation system	Regenerating hCECs	[99]
Mouse	KO	Abolishing tumorigenicity and suppressing CSC phenotype	[93]
iPSCs	KO	Inducing pluripotency	[65]
Human melanoma cells	KO	Demonstrating loss of SOX2 did neither affect melanoma initiation and growth, nor metastasis formation.	[91]
Axolotl	KO	Showing loss of neural stem cell amplification during axolotl tail regeneration	[100]
ESCs	KI	Integrating a suicide gene in-frame to end SOX2 to inhibit differentiation	[101]
ESCs	KO	Establishing a method for conditional KO by using CRISPR-Cas9	[102]
U373MG	Deletion	Deletion of SOX2 regulatory region 2 (SRR2) reduces *SOX2* expression, halts malignant activity of SOX2, and impairs tumor initiation and progression	[85]
NSCs	KI	Monitoring the expression rate of *SOX2* gene	[96]
HEL24.3	KI	Generating hiPSC which contains a SOX2-ntdTomato reporter, to study the expression of *SOX2* in live cells.	[95]

**Table 2:** CRISPR targeting other SOX genes.

Gene Name	Cell Line/ Animal Name	Knock Out/Knock In	Outcome	Refs
*SOX1*	ESCs	KI	For engineering the haploid ES cell genome	[152]
*SOX2, SOX3*	Zebrafish	KO	SOX2 and SOX3 are important for the normal development of otic and epibranchial placodes	[129]
*SOX2, SOX3*	Mice	Gene-swap (KO/KI)	SOX2 and SOX3 proteins are functionally equivalent in brain and testes	[128]
*SOX3*	Zebrafish	KO	SOX3 is important for follicle development and fecundity in zebrafish	[127]
*SOX4*	The bladder cancer cell lines	Knockdown	Decreasing invasive capabilities in bladder cancer	[131]
*SOX5, SOX10*	Medaka, zebrafish	KO	Demonstrating interaction between SOX5 and SOX10	[153]
*SOX6*	K562 cell line	KO	Leading to γ-globin reactivation	[135]
*SOX9*	H9 hESC line	KO	Affecting human lung organoids proliferation and differentiation	[154]
*SOX10*	NSCs	Activation system	Enhancing neural stem cell differentiation	[145]
*SOX10*	Chicken fibroblast cell line	KO	Optimizing of genome editing approach in early chick embryos and perturbing downstream neural crest GRN components	[140]
*SOX10*	hiPSC	KI	Generating neural crest progenitor cells by adding a reporter gene into *SOX10* locus	[141]
*SOX11*	SCs	KO	Haplo insufficiency of SOX11 impairs key processes of human neurodevelopment	[155]
*SOX17*	Mice	KO	SOX17 is critical for embryo implantation and pregnancy	[151]

## Data Availability

Not applicable.

## Abbreviations

CRISPR	Clustered Regularly Interspaced Short Palindromic Repeats
Cas	CRISPR-associated proteins
crRNA	CRISPR RNA
tracrRNA	Trans-activating crRNA
DSB	Double-stranded breaks
sgRNA/gRNA	Single guide-RNA
ZFN	Zinc-finger nuclease
TALEN	Transcription activator-like effector nucleases
PAM	Protospacer Adjacent Motif
SOX	SRY homology box
KO	Knock-out
KI	Knock-in
CSC	Cancer Stem Cell
hESC	Human Embryonic Stem Cell
iC9	iCaspase 9
dCas9	Dead Cas9
TESCO	Testis-specific enhancer of SOX9 core
XYSR	XY sex reversal region
PPAR	Peroxisome proliferator-activated receptor
RYBP	Ring1 and YY1 Binding Protein
CRISPRi	CRISPR inhibitor
PNS	Peripheral nervous system
RAG	Regeneration-associated genes
lncRNA	Long noncoding RNA
OxPhos	Oxidative phosphorylation
